# Transabdominal Ultrasound of the Stomach in Patients with Functional Dyspepsia: A Review

**DOI:** 10.3390/diagnostics14192193

**Published:** 2024-09-30

**Authors:** Sangeeta Roopan, Odd Helge Gilja

**Affiliations:** 1 Department of Clinical Medicine, University of Bergen, 5021 Bergen, Norway; 2 National Centre for Ultrasound in Gastroenterology, Haukeland University Hospital, 5021 Bergen, Norway

**Keywords:** ultrasonography, dyspepsia, stomach, gastric imaging, functional dyspepsia

## Abstract

**Background/Aim:** Dyspepsia is a very common condition worldwide and has an immense impact on quality of life. Functional dyspepsia (FD) is defined by dyspeptic symptoms with the absence of any structural abnormality that can explain the cause. Ultrasonography (US) is a non-invasive imaging modality that can be applied to assess gastric function. The aim of this review paper is to assess how ultrasonography can sort out patients with dyspepsia and help diagnose functional dyspepsia. **Methods:** Using the keywords “functional dyspepsia” and “ultrasonography”, the PubMed database was screened for publications on the use of ultrasonography to identify functional dyspepsia. Afterward, two screening processes were performed to narrow the articles down to a rational number. **Results:** A total of 169 articles were obtained from the literature search, and 31 of these were included after the screening process. Ultrasonography was capable of identifying functional dyspepsia pathophysiology using both two-dimensional (2D) and three-dimensional (3D) ultrasound. **Conclusions:** We conclude that ultrasonography is a non-invasive, safe, low-cost, and widely accessible method that can help diagnose functional dyspepsia through the exclusion of organic dyspepsia and assessing FD pathophysiology. Incorporation of ultrasound in the work-up of patients with functional dyspepsia allows for a sound diagnostic approach and can further improve patient management.

## 1. Introduction

Dyspepsia implies difficult digestion and is characterized by various symptoms from the upper abdomen, such as fullness, discomfort, early satiation, postprandial bloating, belching, nausea, vomiting, or pain [1,2,3,4]. Dyspeptic symptoms were first known to be prevalent in the Western hemisphere; however, due to more studies and awareness, it is now known as a global health issue, influencing 15–20% of the general population [2,4,5,6,7,8]. Its high prevalence and many clinical manifestations make it a common condition seen in clinical practice all over the world [1,6,9].

The etiology of dyspepsia is divided into two categories: organic and functional dyspepsia [1]. Organic dyspepsia is present when there is an underlying disease that can explain the cause of such symptoms, and some examples are peptic ulcers, GERD, malignancy, coeliac disease, pancreatitis disease, and gallbladder disease [1,10]. If a patient has dyspeptic symptoms in the absence of any organic disease, the condition is called functional dyspepsia [1,3,4,11]. The majority of dyspeptic patients fall into the latter category [1,4].

To diagnose functional dyspepsia, it is important to exclude organic causes [6]. This is usually performed through clinical examination, lab work, and an upper gastrointestinal endoscopy [1,6]. Endoscopy findings are often negative in dyspeptic symptoms, thus suggesting the diagnosis of functional dyspepsia [6,7,10].

The Rome criteria classify functional dyspepsia as having one or more of the following cardinal symptoms: postprandial fullness; early satiation; epigastric pain; or epigastric burning [1,11]. In addition, there should be no evidence of structural disease, including a negative endoscopy, that could explain such symptoms [11,12]. Furthermore, these criteria must be fulfilled for the last 3 months, with symptom onset at least 6 months prior to diagnosis [11,12]. The Rome IV criteria also subdivide functional dyspepsia into two groups, postprandial distress syndrome (PDS) and epigastric pain syndrome (EPS), depending on which symptoms are most dominant [1,5,12,13].

Once the diagnosis of functional dyspepsia is finalized, patients are sometimes offered further examination. One could ask why additional examinations are proposed when there are no findings on endoscopy or other clinical tests. Some FD patients find it difficult to accept a diagnosis that has no identifiable cause, and in the hope of finding any pathology that could explain such symptoms, further investigations are often demanded by the patients [6]. Throughout the years, research has led to the discovery of several pathophysiological mechanisms unique to functional dyspepsia that could possibly explain symptoms. These pathophysiological mechanisms seem to be a combination of sensory, motor, and psychological disturbances [5,14].

### 1.1. Pathogenesis of Functional Dyspepsia

Functional dyspepsia has a heterogeneous pathophysiology, and symptoms are shown to mainly revolve around mealtimes [15,16]. During meal ingestion, the proximal stomach accommodates to create a reservoir for the meal [1,14]. This happens in two phases: the receptive and adaptive phases [1,13,17]. Receptive relaxation is the dilation of the proximal stomach in response to food that passes down the pharynx/esophagus, which aims to prepare the stomach for food ingestion [1,13,18,19]. Adaptive relaxation is accommodation in regard to an increase in nutrient volume through mechanical distension of the proximal stomach [1,13].

While the proximal stomach is responsible for storing food, the antrum and the pyloric region are responsible for the mixing, grinding, and emptying of the meal [18]. This is performed through phasic contractions that occur three times per minute in the fed state; these start from the mid-stomach and sweep slowly toward the pyloric region [1]. In FD patients, abnormalities of these gastric motor functions are identifiable, and the most documented are impaired gastric accommodation, delayed gastric emptying, and visceral hypersensitivity [3,5,12,14,15].

Impaired gastric accommodation has been identified in 40% of FD patients [11,12,13]. Patients with impaired gastric accommodation lack proximal stomach dilation in response to a meal [1,11,12]. This leads to an increase in intragastric pressure and possible dyspeptic symptoms [1,11,15].

A wide antrum during fasting and postprandial state has been observed in many FD patients [1,20,21,22,23]. The antroduodenal region is known to have other disturbances as well, such as delayed gastric emptying and antrum dysmotility [1,20]. The former has been identified in approximately 30% of FD patients and is described by a prolonged emptying of food contents from the antrum into the duodenum [12,24]. There are still questions raised about whether antrum dilation is a consequence of impaired gastric accommodation, delayed gastric emptying, true antral dysmotility, or all [1]. Some studies suggest that due to the higher prevalence of impaired gastric accommodation, antrum distension is a secondary cause due to lack of proximal relaxation [10]. Other studies state that antrum dysmotility and wide antrum are linked together [20,24,25]. Some studies also suggest a possible relationship between antral dysmotility and delayed gastric emptying, while others have not [2,5,26].

Another important pathophysiological mechanism found in FD patients is visceral hypersensitivity [1,21]. Visceral hypersensitivity is defined as abnormally enhanced perception of visceral stimuli and occurs in about 33% of FD patients [1,12]. Several hypotheses explain visceral hypersensitivity as an aftermath of defects in the brain–gut axis [1]. This is a topic of interest in functional dyspepsia due to the high prevalence of psychological comorbidity amongst FD patients [1,6]. The most dominating comorbidities are anxiety, depression, headache, myalgia, fibromyalgia, asthenia, irritable bowel syndrome (IBS), and chronic fatigue syndrome [1,6,21]. Whether these psychological disorders are independent manifestations or modulate the brain–gut axis, thus influencing pathophysiology in functional dyspepsia, is a matter of great debate [1,21].

There are several different modalities applied to assess gastric function in FD patients: the barostat; single-photon emission computed tomography (SPECT); magnetic resonance imaging (MRI); scintigraphy; ultrasonography; and drink tests [13,14,15]. Each has its own advantages and disadvantages and assesses different aspects of gastric and non-organic pathophysiology.

### 1.2. Imaging Techniques

Scintigraphy is a non-invasive method that has long been the gold standard for assessing gastric emptying and intragastric distribution through the ingestion of a radiolabelled meal [4,13,25,27]. It has its limitations, such as poor image resolution and requirement of specific radiolabelled meals [10,13,27]. Scintigraphy is also expensive and exposes the patient to radiation, which limits its use in clinical practice [10,13,25,27].

The barostat is well-suited to identify impaired gastric accommodation [5,12,13,14,15]. The procedure is invasive as it requires inserting a polyethylene balloon in the gastric fundus through oral intubation [11,13,14]. The barostat is not widely available, highly invasive, and uncomfortable for patients, which can lead to biased measurements of tone and sensation [13,14,15]. It is time-consuming and requires expertise, which is only present at a few tertiary centers [14,15].

SPECT (Single-Photon Emission Computed Tomography) scanning is a non-invasive technique that has been used to assess gastric accommodation [15]. The procedure requires intravenous administering of Tc-99 pertechnetate and a SPECT gamma camera for imaging [13,14,15]. This method exposes the patient to radiation, the equipment is not widely available, and it is a high-cost procedure [13,14,15]. SPECT also requires the supine position of the patient, which, in turn, does not resemble real-life meal ingestion [14,15].

MRI is a non-invasive technique that is mainly applied to estimate intragastric meal distribution and gastric emptying [14,15]. It has also been applied to assess gastric accommodation through 3D reconstruction of the total gastric volume [14,15,19]. The benefit of applying MRI is that it is radiation-free, safe, and capable of differentiating gas from solid food [13,14,15]. However, MRI is time-consuming, expensive, and requires the supine position of the patient, similar to SPECT [13,14,15]. In addition, this method requires specific test meals labeled with gadolinium, which also contributes to low accessibility [13].

Functional dyspepsia is a condition with many challenges due to its heterogeneous clinical presentation, uncertain pathogenesis, and need for a suitable modality that can assess most of the various gastric motor functions in one session [6]. This leads to a significant reduction in quality of life for these patients [7,12,18]. Considering its high prevalence in society and the burden it imposes on patients, functional dyspepsia is a condition that is in need of improved imaging methodology.

Ultrasonography is an imaging modality that is widely used in many aspects of the medical field and has proved to be useful in supporting many important interventions [14]. Its use in patients with dyspepsia and functional dyspepsia has been well documented, but there is still considerable underuse of ultrasound methods worldwide. In this review, we aim to assess how ultrasonography can sort out patients with dyspepsia and help diagnose functional dyspepsia.

## 2. Materials and Methods

**Figure A1:**
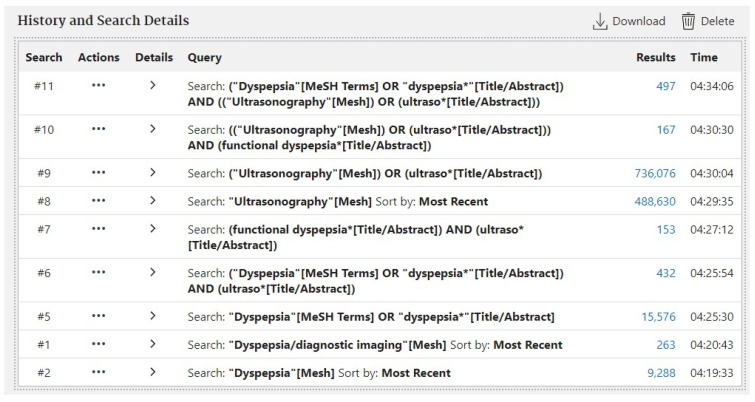
This figure presents the finalized search conducted on the 20 September 2023, applying the search words “functional dyspepsia” and “ultrasonography” in the PubMed database. The number of articles retrieved was 167. Another search was performed on the 24 February 2024 using the same literature search, which found one more article that was published in December 2023. This article, along with another one advised by the supervisor, was included, which increased the total number of articles to 169.

Articles were retrieved from PubMed, and the time frame was set to 1 January 1980–31 December 2023. The search was performed using the PubMed Advanced Search function within the field Title/Abstract. The first search included keywords “ultrasonography” and “dyspepsia” and was performed in August 2023. It retrieved 392 articles, which was too extensive for this review. Consequently, to reduce the number of articles, the topic of this paper was modified from dyspepsia and ultrasonography to functional dyspepsia and ultrasonography. Various search terms were tested out, such as “gastric emptying”, “gastric accommodation”, and “functional dyspepsia”, in combination with “ultrasonography”. “Gastric emptying” and “Gastric accommodation” retrieved n = 608 and n = 46 papers, respectively. The final search was performed on 20 September 2023 and included the words “ultrasonography” and “functional dyspepsia”, which yielded 167 articles for the final analysis. “Ultrasonography” was searched through both free text, using the phrase “ultraso*”, and through MeSH “Ultrasonography”. This was then paired with “functional dyspepsia” in free text. The final search can be viewed in Figure A1.

A second search was performed (with the same search phrases) on 22 February 2024. This was to guarantee the inclusion of all articles from the specified time frame. The search returned an article published in December 2023, which was included. Yet another article was added later due to its high relevance for the three-dimensional ultrasonography technique.

After the articles were obtained, two screening processes took place: one by reviewing the title and abstract and the second by reading full articles. Specific criteria were made before the screening process, and these are presented in Table 1. The inclusion criteria focused mainly on English articles that included adults with functional dyspepsia and that had applied ultrasound to identify functional dyspepsia pathophysiology. After retrieving the appropriate papers, a quality assessment was performed. The quality assessment questions are shown in Table 2. Furthermore, the journals were searched and rated through the *Norwegian Register of Scientific Journals, Series, and publishers* based on which scientific level they had, and the majority had a scientific level of 1 (the highest being level 2). After the quality assessment of the articles, data were extracted from the scientific studies that were directly related to functional dyspepsia and ultrasound. Author, year, country, aim, ultrasound equipment, transducer location, ultrasound measurements, test meals, and patient position were data extracted from these articles.

## 3. Results

**Figure 1:**
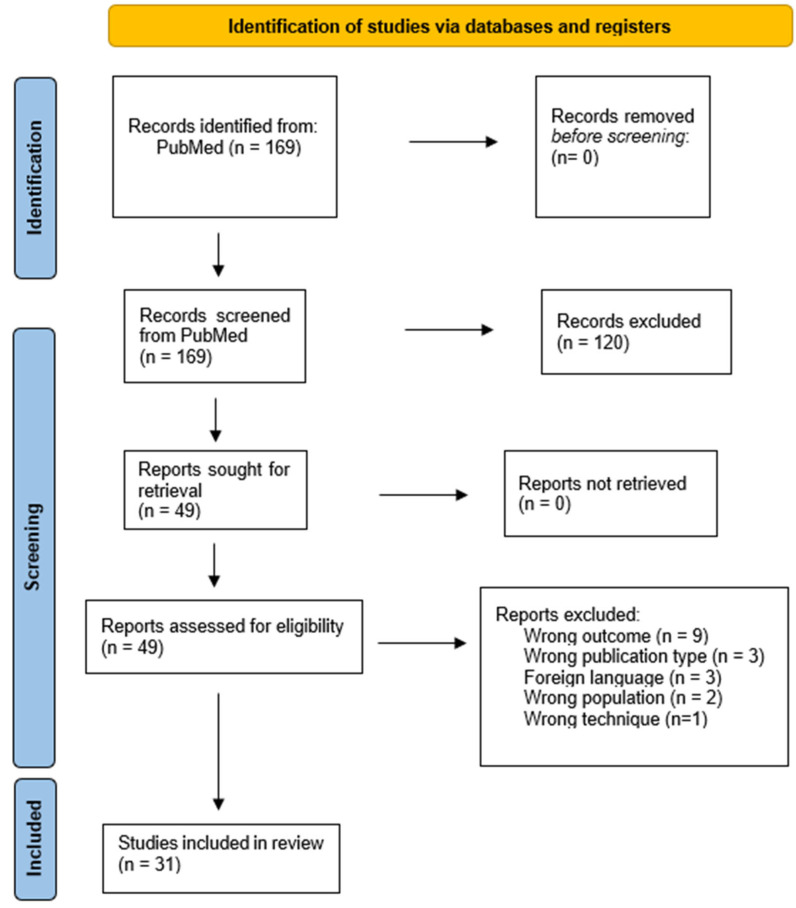
PRISMA diagram that shows the whole process of how many articles were obtained from PubMed using the search words “functional dyspepsia” and “ultrasonography”. A total of 169 articles were retrieved from PubMed. Two screening processes took place; the first based on title/abstract, and the second based on a read of the full article. The total number of articles included in this review was 31.

**Figure 2:**
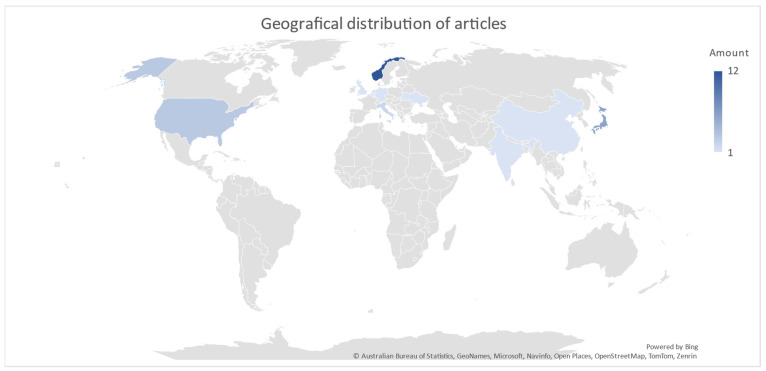
This map shows the geographical distribution of the articles included in this paper. Norway and Japan had the most articles on the combination of search terms “ultrasonography” and “functional dyspepsia”.

A total of 167 articles were obtained from the literature search. After adding the article published in December 2023 and another highly relevant article on three-dimensional ultrasonography, the total amount of articles included in this paper was 169. Through the first screening, 120 titles/abstracts did not meet the inclusion criteria. The remaining 49 articles were read in full length, and 18 did not meet the inclusion criteria. Through the two screening processes, 31 articles remained. A thorough overview of the screening process is shown in the PRISMA diagram (Figure 1). The majority of articles included in this paper originate from Norway and Japan (Figure 2). Out of 31 articles, 25 are scientific studies, and the remaining 6 are review papers used for background information. Out of 25 scientific articles, 19 are directly related to functional dyspepsia pathophysiology and ultrasonography, and data extraction was conducted on these. The data extracted are presented in Table A1. The other six scientific articles were relevant to ultrasound technique and background information.

### 3.1. The Use of Ultrasonography to Assess FD Pathophysiology

Ultrasonography is a non-invasive, radiation-free imaging modality that uses sound waves to reveal underlying anatomy and pathology [2,5,15,22,25]. Ultrasonography has the advantage of being available all over the world, as well as in underserved corners of the globe. The use of ultrasonography is not foreign to functional dyspepsia, and this is shown through the various studies where ultrasonography has been evaluated as a diagnostic tool. FD was defined based on the Rome classification system or using similar criteria regarding dyspeptic symptoms with the exclusion of organic disease through at least a negative upper gastrointestinal endoscopy. These studies applied ultrasonography to assess various pathophysiological abnormalities unique to functional dyspepsia, and there were most often two specific parts of the stomach analyzed: the proximal and distal gastric compartments.

### 3.2. Proximal Stomach

Ultrasonography was made available to assess the proximal stomach, and the goal was to identify impaired gastric accommodation, which is frequently seen in FD patients. In principle, there were two ways to estimate gastric accommodation by ultrasound: either by calculating changes in area/diameter or volume of the proximal stomach. Area changes were an indirect measurement, while volume changes were a direct measurement of proximal gastric volume changes. Using 2D ultrasound, the majority of studies included in this paper evaluated gastric accommodation based on changes in the proximal stomach area, and in the literature, there are two different techniques applied to obtain these measurements.

**Figure 3:**
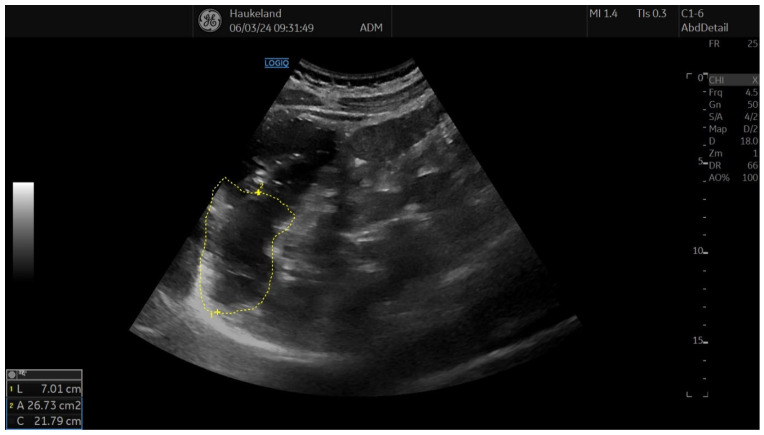
This image shows a measurement of proximal gastric area (PGA) using 2D-US in sagittal section, and this is used to assess gastric accommodation. PGA was obtained by placing the transducer longitudinally under the left subcostal margin and tilting it cranially in the long axial direction. Internal landmarks to look for were the left kidney in the longitudinal projection, the left lobe of the liver, and the tail of the pancreas. PGA was calculated by tracing along the luminal echogenic surface, which corresponded to the interface between the gastric liquid and the mucosa of the gastric wall. This was performed from the top margin of the fundus to 7 cm inferiorly along the axis of the proximal stomach. This image was taken after meat soup ingestion as part of the ultrasound meal accommodation test (UMAT) that is regularly performed at Haukeland University Hospital, Bergen, Norway.

**Figure 4:**
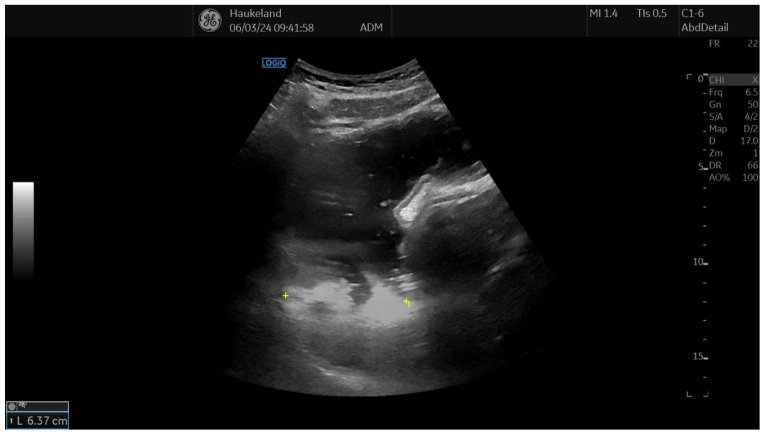
This image shows the measurement of the maximal proximal gastric diameter (MPGD) using 2D-US, which is used to assess gastric accommodation. MPGD was obtained by rotating the transducer 90° clockwise after holding it in the position to obtain PGA and tilting it cranially in the short axial direction. Internal landmarks to look for were the left hemidiaphragm, top margin of the fundus, and liver parenchyma. The diameter was measured within the 7 cm long axis of the proximal stomach. This image was taken after meat soup ingestion as part of the UMAT at Haukeland University Hospital, Bergen, Norway.

The most common method applied to assess proximal gastric accommodation was by assessing changes in proximal gastric area (PGA) and maximal proximal gastric diameter (MPGD). Changes in PGA and MPGD after meal ingestion were used as indirect measurements of proximal gastric volume changes, thus reflecting gastric accommodation. To obtain PGA, the transducer was placed in the epigastrium longitudinally under the left subcostal margin and tilted cranially in the long axial direction [11,12,18,22]. This allowed for a visualization of a sagittal section of the proximal stomach, and internal landmarks were the left kidney in the longitudinal projection, the left lobe of the liver, and the tail of the pancreas [11,12,18,22]. On the sagittal section, PGA was calculated by tracing along the luminal echogenic surface, which corresponded to the interface between the gastric liquid and the mucosa of the gastric wall (Figure 3) [11,12]. This was performed from the top margin of the fundus to 7 cm inferiorly along the axis of the proximal stomach (Figure 3) [11,12,18,22]. MPGD was obtained from an oblique frontal section (transverse section), which was achieved by rotating the probe 90° clockwise and tilting it cranially in the short axial direction [11,12,18,22]. The landmarks to look for in the transverse section were the left hemidiaphragm, the top margin of the fundus, and the liver parenchyma [11,12,18,22]. The diameter was measured within the 7 cm long axis of the proximal stomach (Figure 4) [18,22].

By assessing the sagittal area and oblique frontal diameter measurements after meal ingestion, the accommodation reflex could be calculated [11,12,18]. This 2D ultrasound method was first developed by Gilja et al., and its use has spread out to other countries around the world [11]. Other papers also applied PGA and MPGD to estimate proximal gastric volume (PGV) by multiplying them together [14,15,18,22]. PGV was then used to estimate the proximal gastric emptying rate, which described how fast the volume decreased per minute after meal ingestion [18,22]. This was also utilized as a measure of gastric accommodation.

The majority of the studies using 2D ultrasound to assess proximal gastric content calculated PGA and MPGD to determine impaired gastric accommodation. However, another method was applied to obtain the proximal gastric area, and this was utilized mainly in Japan. The Japanese studies applied a different visualization position of the proximal stomach. The ultrasound transducer was placed on the left side of the abdomen in the 10th intercostal space using the spleen as an acoustic window [3,28]. This method yielded a cross-sectional image of the proximal stomach [3,17,28]. Using this scanning position, the cross-sectional area of the proximal stomach was calculated by tracing the mucosal surface of the gastric lumen [3,17,28]. Looking at changes in the area before and after meal ingestion allowed for the assessment of gastric accommodation [3,28].

Even though the studies used different techniques to evaluate the proximal gastric area, their examination procedures were similar. Patients were required to undergo an overnight fast, preferably seated, leaning slightly backward, while they ingested a test meal that was consumed over a short amount of time [11,12,18]. What differed from the examination procedures was the ultrasound scanners, probes, and test meals applied. Norwegian studies combined ultrasonography with a low-caloric (20 kcal) meat soup (500 mL), while the Japanese studies combined it with water (400 mL or 800 mL) [3,6,17,18,28]. The other studies applied variants of liquid test meals. All ultrasound scanners differed, and the transducers ranged from 2–5 MHz [3,12,18]. In addition, the Japanese studies had the patients lying in the supine position in order to achieve an optimal cross-sectional view of the proximal stomach [3,17,28].

Despite all the various techniques used to assess gastric accommodation, the results from these studies were often in agreement. PGA, MPGD, and cross-sectional proximal gastric area proved to be smaller postprandially in FD patients compared to healthy controls [3,12,17,18,28]. PGV was applied to measure the proximal gastric emptying rate, and in FD patients, the proximal gastric emptying rate was higher when compared to healthy controls [18,22]. The results show that after meal ingestion, meal contents do not reside in the proximal reservoir and instead are rapidly moved to the antrum, giving rise to antral distension and more symptoms. These findings confirm that impaired gastric accommodation is a significant cause of symptoms found in many patients with functional dyspepsia.

### 3.3. Distal Stomach

**Figure 5:**
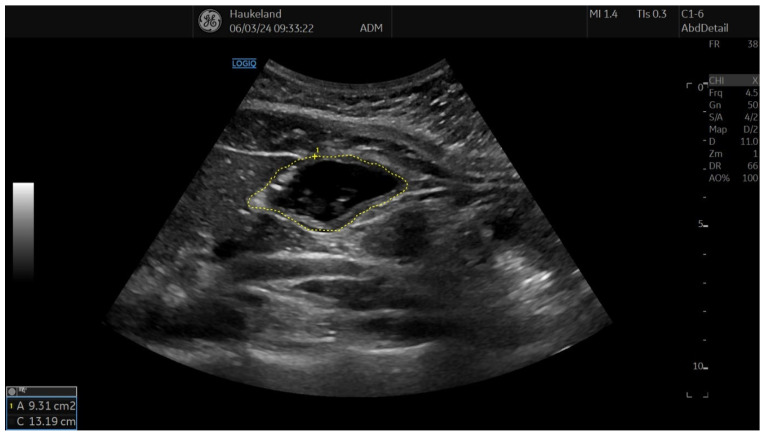
This image shows the cross-sectional view of the antrum using 2D-US and is used to assess gastric emptying and antrum motility. Cross-sectional view of the antrum was obtained by placing the transducer vertically in the epigastrium to achieve a sagittal plane with the superior mesenteric vein and aorta serving as internal landmarks. Using this image, the cross-sectional antral area was measured by tracing around the proper muscle layer of the antral wall. This image was taken after meat soup ingestion as part of the UMAT at Haukeland University Hospital, Bergen, Norway.

The distal part of the stomach is defined as the antrum region and has been shown to have several disturbed mechanisms in functional dyspepsia patients, such as delayed gastric emptying and antroduodenal dysmotility. To view the antrum adequately, the following approach has been used: the ultrasound probe was placed vertically in the epigastrium to achieve a sagittal plane, with the superior mesenteric vein and aorta serving as internal landmarks [20,22,24,25,26,29]. In this projection, an image of the cross-section of the antrum was achieved [22,25,26,29]. They were different approaches to calculating the cross-sectional antral area, and they differed geographically. Norwegian studies obtained antrum area by tracing around the proper muscle layer of the antral wall (Figure 5) [5,20,27]. Japanese studies traced the antral mucosa [26,29]. Bolondi et al. calculated the cross-sectional antral area by measuring longitudinal and anteroposterior diameters from the outer profile of the antrum wall and applying it in a dedicated formula [25]. Cross-sectional antral area calculations were used to identify both antral distensions, delayed gastric emptying, and antral dysmotility [22].

Delayed gastric emptying has been one of the most frequent pathophysiological mechanisms studied with ultrasonography with regard to functional dyspepsia. There are two main ways to evaluate gastric emptying: by calculating gastric emptying rate (GER) or gastric emptying time (GET). The majority of studies calculated the gastric emptying rate, and this was based on assessing the rate of decrease in the antral area after meal ingestion [22,26,29]. This was a prominent method that was especially used in the Japanese and Norwegian studies. Another study by Ahluwalia et al. calculated gastric emptying rate by measuring the decrease in relaxed antral circumference rather than area [2].

The gastric emptying time was a common method applied in Italian and German studies [24,25,30]. This method was executed by calculating how much time it took until the antral area reached basal values (fasting antral area) [24,25]. The study by Bolondi et al. also attempted to calculate gastric emptying time through changes in antropyloric volume computed by a dedicated formula based on 2D ultrasound measurements [25]. Antropyloric volume was calculated until it returned to basal values, and the time used was reported as the gastric emptying time [25]. Antropyloric volume was estimated using a formula based on longitudinal and anteroposterior diameters at three different levels and the length of the antropyloric region [25].

Studies on gastric emptying rate and gastric emptying time were both able to show that gastric emptying was delayed in FD patients, showing slower gastric emptying rates and longer gastric emptying time [24,25,29,30].

The cross-sectional area of the antrum was also used to examine the motility characteristics of the antroduodenal region. One common method that was applied in the included studies was to use the frequency and amplitude of antral contractions to assess contractility. The frequency multiplied by the amplitude was calculated as the motility index (MI), and this was shown to be lower in FD patients, suggesting antrum hypomotility [22,26,29]. According to Haruma et al. and Kusunoki et al., antrum hypomotility was more significant during the early postprandial phase [26,29]. Berstad et al. also found a wider fasting and postprandial antrum in FD patients compared to healthy volunteers, and this was also found in other studies [22,24,25]. Ahluwalia et al. showed that antrum width varied within a group of FD patients, suggesting that a wide antrum might not be a consistent finding in all patients [2,10]. A more advanced method was developed to assess antrum motility, and this is based on antral contraction strain measurements obtained by ultrasound strain rate imaging [5]. This method was based on using ultrasound Doppler to assess the deformation of the antrum wall during contractions [5]. This method demonstrated such a high level of detail that the outer longitudinal smooth muscle layer of the gastric wall could be distinguished from the inner circular smooth muscle layer, showing distinct different patterns of motility [5].

Studies that focused on antroduodenal characteristics were undertaken using similar examination procedures. Patients were required to fast overnight and sit slightly leaning backward while ingesting a test meal over a short period of time [2,26,29]. Meal tests used in these studies varied between meat soups, nutrient-test meals, water, and solid meals [2,5,25,30]. The ultrasound scanners that were used were from different vendors, and they applied various ultrasound probes ranging from 3 to 7 MHz [24,25,29].

### 3.4. 3D-Ultrasonography

The methods described above for imaging both the proximal and distal parts of the stomach were based on 2D ultrasound. Two-dimensional ultrasound has shown its capability to identify functional dyspepsia pathology, but some inherent limitations led to the development of three-dimensional ultrasound.

Two procedures were developed in order to estimate gastric volume using three-dimensional ultrasound. The first method explored calculated volumes based on a tilting movement of the transducer [22,23]. The transducer was coupled to a motor device that held the probe in a fixed position while enabling an accurate motorized movement in a tilting fashion [19,23,27]. This movement was limited to a 90° angle and allowed for volume estimation of a fan-like region [23,27]. From this fan-like acquisition, sequential 2D images were gathered and transferred to a graphic workstation for reconstruction into 3D images that allowed for volume estimation [23,27]. Using this tilting technique, volume estimation was only possible to obtain for one limited area because of the fixed transducer position, and it worked well for the antrum only [23,27]. This posed challenges for the estimation of larger gastric volumes, such as total gastric volume [10,27].

With this limitation in mind, a new technique was developed utilizing a magneto-based system, which could enable greater flexibility in handling the transducer [10,19,27]. The magneto-based system incorporated a transmitter and a sensor, which made it possible to move the transducer freely while registering the exact position and orientation of the transducer in space at all times [27,31]. The transmitter was placed behind the patient’s back and generated a magnetic field while the magnetic sensor was attached firmly on top of the ultrasound probe [27,31]. A long, continuous sweep was performed with the transducer from the fundus to the pylorus, thus acquiring 2D images in multiple sagittal sections [31]. Regions of interest were manually outlined on the sagittal sections by tracing the gastric mucosa [15,27,31]. Using a dedicated software application, the sagittal outlines, together with data on position and orientation, made it possible to reconstruct the 3D images and subsequently calculate the total gastric volume [15,27,31].

Separate gastric volumes could be calculated in the postprocessing in two different ways based on where the dividing plane was positioned in the stomach. The study by Mundt et al. estimated PGV and distal gastric volume by dividing the total gastric volume into two different sections [31]. PGV was defined by a dividing plane 10 cm downwards from the diaphragm, perpendicular to the longitudinal axis of the stomach [31]. The distal volume was defined by a plane perpendicular to the antral axis where the liver, superior mesenteric vein, and aorta were seen simultaneously [31]. By subtracting PGV and antral volume from TGV, Mundt et al. managed to calculate the corpus volume as well [31]. To assess gastric accommodation, Mundt et al. also calculated the ratios between TGV and PGV [31]. Gilja et al. calculated PGV and distal gastric volume according to anatomy by creating a division plane vertically at the angular incisure [27]. Furthermore, Gilja et al. also estimated intragastric meal distribution by dividing the proximal gastric volume by the distal gastric volume [27].

Three-dimensional ultrasound has shown that FD patients had smaller PGV after meal ingestion [11,31]. Berstad et al. and Mundt et al. found larger antrum volumes in their studies, while Gilja et al. found no significant differences [10,22,23,31]. Furthermore, Mundt et al. reported no difference in TGV between patients and controls [31]. However, intragastric maldistribution of food contents was reported, implying a rapid shift of food contents from the proximal to the distal part of the stomach [31].

### 3.5. Visceral Hypersensitivity

The articles that have studied pathologies of the proximal and distal parts of the stomach have often simultaneously evaluated symptoms before and after meal ingestion in order to assess visceral hypersensitivity. There were two different symptom scales used in the various studies: the Visual analog scale (VAS) and the Likert scale [3,6,7,18]. The studies showed that FD patients had more symptoms after meal ingestion than healthy controls, suggesting visceral hypersensitivity to be a key factor in symptom generation in these patients [3,6,7,18].

To sum up, the articles included in this review paper applied both 2D ultrasound and 3D ultrasound to assess functional dyspepsia pathophysiology. Ultrasonography was reported to identify a smaller proximal stomach postprandially, change in intragastric distribution, delayed gastric emptying, antrum dysmotility, and visceral hypersensitivity in FD patients compared to healthy controls (HC).

## 4. Discussion

In this paper, a summary of functional dyspepsia pathogenesis and diagnostic procedures is given. Furthermore, an overview of the use of ultrasonography to look for FD pathophysiology is provided. It is evident that ultrasonography can be applied in many different ways to manage patients with functional dyspepsia. Ultrasonography, being non-invasive and safe to use, has many advantages that could promote improved work-up of patients with FD.

### 4.1. Diagnostic Benefits of Ultrasonography

Ultrasonography is a tolerable and radiation-free modality that can contribute to a more comfortable experience for patients with dyspepsia [13,14,22,29]. A comfortable examination is in itself a huge benefit for patients, particularly if repeated examinations are required. With ultrasonography, gastric physiology is left undisturbed, and long-term radiation burdens are avoided [13,18]. In addition, patients would not have to lie in a very tight space with noise-making MR machines that could be frightening or trigger claustrophobia [6,19]. A calm environment also helps in avoiding biased results regarding gastric tone and symptom analysis, as FD patients are known to be sensitive to psychological stress [19]. Only one-fourth of dyspeptic patients are known to seek medical care regarding their symptoms, and if ultrasonography was incorporated, it could possibly encourage patients to seek medical attention more frequently, being a non-invasive procedure [1].

Ultrasonography shows real-time images with high temporal resolution, thus providing physicians and patients with immediate results [2,6,19,27]. This allows for dialogue between physician and patient throughout the examination about relevant findings [6]. A thorough explanation of the underlying pathology and presentation of relevant findings could provide comfort for many patients. This may provide a better understanding of their condition, which could potentially increase their acceptability and improvement of their condition [6].

Compared to other modalities, like MR and scintigraphy, ultrasonography is a low-cost imaging method [5,11,13,14]. As mentioned above, functional dyspepsia implies a huge economic burden on patients and society, and a study by Lacy et al. looked at how much the actual economic impact was. The authors looked into expenses spent on medical visits, medical testing, surgery, treatment, and indirect costs related to work productivity [8]. Adding these together, the cost per patient was USD 1595, which sums up the economic strain FD patients have, and it also shows how resource-demanding functional dyspepsia is for the health care system [6,8].

This study also presented the separate costs for the various medical tests included, and ultrasonography was shown to be the cheapest medical test ($84) after bloodwork, 24-h pH-testing, and x-ray [8]. As mentioned before, upper gastrointestinal endoscopy is needed to exclude any organic pathology, and replacing endoscopy with ultrasound is highly unlikely due to the increased accuracy endoscopy holds regarding superficial mucosal lesions in the GI tract. However, ultrasonography can exclude organic pathology outside the GI tract [6]. Ultrasound is regularly used to search for organic diseases in some centers around the world, e.g., at Haukeland University Hospital, Bergen, Norway [6]. FD patients are scanned for organic diseases using the systematic 6+ method prior to the ultrasound soup test (UMAT) assessment of functional dyspepsia pathophysiology [6]. The 6 + method provides a systematic scan of the liver, gallbladder, biliary tract, spleen, pancreas, kidneys, urinary bladder, large vessels, and GI tract in search of abnormalities [19]. This could reduce the costs by removing the need to use other, more expensive imaging modalities in order to rule out organic pathology.

Not only can ultrasound help reduce costs when excluding organic pathology, but it can also help reduce costs by replacing other modalities that have been used to assess functional dyspepsia pathophysiology. Furthermore, ultrasonography has the capability of viewing several gastric motility parameters simultaneously, and to date, it is the only modality of the ones available that can view the most parameters of gastric motility [14,19]. With ultrasonography, gastric emptying, intragastric distribution, strain of the gastric wall, gastric accommodation, antral motility, and visceral hypersensitivity can be assessed during the same procedure [19]. This eliminates the need for different modalities to assess different pathophysiological mechanisms, thus reducing expenses. Furthermore, having one modality is more convenient for the patient than having to undergo several different diagnostic procedures.

Even though ultrasonography makes the examination procedure much easier and more comfortable, it still requires a certain level of expertise, which questions its accessibility in clinical practice [13]. Ultrasonography is a fairly operator-dependent modality, and therefore, the operator must have sonographic skills and experience to interpret the visual information displayed [14,18,22]. Other limitations of the ultrasound method are the inability to take gastric secretion and bile reflux into account when estimating gastric emptying. Furthermore, this method performs best with liquid meals, not solid food. This imposes a challenge for its incorporation in clinical practice, but compared to other modalities used in functional dyspepsia diagnostics, ultrasonography is widely accessible, and training programs are available [5,12,14]. Almost every hospital around the globe has an ultrasound scanner, whereas SPECT, MRI, and scintigraphy are rarely seen in underserved areas of the world. Compared to the other modalities, ultrasound’s familiarity and accessibility could lower the threshold for applying it in dyspepsia diagnostics in primary health care centers as well as in tertiary hospitals.

Another reason that makes ultrasonography accessible is that it can be performed with a wide range of ultrasound scanners from low-end to high-end machines. The various studies included had applied different types of scanners and were able to report the same type of measurements, and studies achieved comparable results. As for the probes, transducer frequency between 3 and 5 MHz was the most common frequency applied and seemed the most optimal. This wide range also reflects ultrasound’s great flexibility in use. Furthermore, ultrasonography can be combined with any kind of test meal, making it more favorable than the other modalities that have to use specific test meals [25].

Ultrasonography has all these advantages; however, is it sufficient compared to other modalities so that it can possibly replace them when determining FD pathophysiology?

The studies included have been able to assess the proximal stomach with conventional ultrasonography. This was performed in two different ways: either by placing the transducer in the epigastrium or by the 10th intercostal space left of the abdomen. Once the proximal stomach was in view, measurements were followed up after meal ingestion. PGA and MPGD were estimated from visualization through the epigastrium, and cross-sectional area was estimated from visualization through the intercostal space. Gastric accommodation was assessed by looking at PGA, MPGD, and cross-sectional area changes postprandially. MPGD measurements have varied in outcome amongst the studies included. According to Gilja et al., it was shown to be smaller in FD patients, but Fan et al. found no significant difference [11,18]. Fan et al. acknowledged the differences between calculating PGA and MPGD measurements by stating that PGA was achieved more easily [11]. They found area measurements to work better than diameter when estimating the size of organs with irregular contours, such as the stomach [11]. PGA was also shown to be less operator-dependent and simple to measure, and it was found to correlate well with gastric volume measurements with the barostat [11,12]. To sum up, it seems that PGA is well-validated when used to identify gastric accommodation in functional dyspepsia, and this is supported by the agreement found among the studies.

Some articles have proposed various reasons for why there might be difficulty in visualizing the proximal stomach. It has been suggested that the presence of air pockets, certain anatomical structures such as costal margins, and obese patients could impose challenges [10,13,14,15,22]. Some studies decided to assess how difficult intragastric air was by grading each image from one to three based on whether image quality was affected by air or not [11,18]. Fan et al. showed that most of the images had no significant impact from intragastric air, and none were excluded due to air pockets [11]. Another study also avoided excluding any images due to intragastric air [18,22]. The latter study was able to reduce air pockets by having the patient sit slightly leaning back so that gas could collect in the anterior part of the fundus and not disrupt the measurements [22]. Other ways to improve image quality by reducing air pockets was by first cooking the meal and then cooling the meal before having the patient ingest it [5,12,18,27]. Furthermore, having the patient fasting overnight also helped reduce the amount of air in the GI tract [11,12]. When it comes to visualization of the antrum, according to Bolondi et al., the gastric antrum was easily recognizable and measurable, even in obese patients [25].

Gastric antrum studies only applied one method to evaluate the distal stomach. Ultrasound was used to calculate cross-sectional antral area measurements, and these were used to assess both gastric emptying and antral motility. The studies varied in how they traced the antrum area, between tracing the mucosal surface or further outward on the antrum wall. According to Bolondi et al., tracing the inner side of the wall is difficult due to narrow antral lumens in fasting patients and difficulty in outlining the inner gastric wall [25]. This may suggest an advantage for outlining the antral area using outer profiles of the antrum wall.

Calculating GET based only on antrum measurements was found to correlate well with scintigraphy measurements that calculated GET from total gastric emptying [22,24]. When estimating antrum motility, ultrasonography was shown to be superior to manometry [5,22]. A study conducted by Hveem et al. compared ultrasonographic antral area measurements to the amount of fasting gastric juice aspirated. Many FD patients are known to have a wide fasting antrum, and according to Hveem et al., this was due to the increased fasting gastric juice content in FD patients [20]. This study showed that volumes of aspirated gastric juice correlated well with the antral area measured by ultrasound [20]. This confirmed that the use of cross-sectional area measurements of the antrum was representative of the assessment of antrum volume.

The majority of studies on proximal and distal parts of the stomach had the patient sitting in an upright position. Other modalities, such as computed tomography or MRI and Japanese studies on gastric accommodation, required a supine position of the patient. Having the patient in a supine position eliminated the resemblance of an actual meal and, more importantly, eliminated gravity’s influence on the meal distribution in the stomach [6,14,19]. Gravity plays a role in the propulsion of gastric contents, and patients should, therefore, be examined in a sitting position, which is easily achieved with ultrasonography [11,19,25]. Antrum visualization was also easier if the patient sat in an upright position, and this also allowed for better detection of food contents in the stomach [25]. Ultrasound’s capability to detect the presence or absence of food remnants was shown to be satisfactory when validated against an X-ray [25]. To sum up, it seems that the most favorable way to identify the proximal and distal stomach is through an upright position, thus using PGA, MPGD, and cross-sectional antral area measurements for assessment of impaired gastric accommodation and antrum motor functions, respectively.

The proximal and distal stomach areas were not only visualized by conventional ultrasonography but also by 3D ultrasound. A 2D ultrasound volume estimation has been applied to assess impaired gastric accommodation, delayed gastric emptying, and antral dysmotility. However, volume estimation based on 2D ultrasound acquisition is challenging and can easily lead to errors. This is mainly due to the need for geometric assumptions of the anatomy when calculating volumes [10,11,22,23]. Three-dimensional ultrasound, on the other hand, was shown to calculate volumes more accurately and eliminate the need for landmarks and geometrical assumptions [11,27].

Both 3D ultrasound techniques mentioned previously were validated in vitro and in vivo [19,27]. The tilting device was evaluated on phantoms and abdominal organs in vitro, which yielded high accuracy and precision [10,22,23]. It was also compared with MRI in vivo and showed good agreement [10,19,22,23]. The magneto-based system was validated in vitro (on porcine stomach) and in vivo with high accuracy as well [19,27]. This method was also validated both concerning its precision in the location of specific points in space and its accuracy in volume estimation [10,27].

When comparing 3D-volume calculations with other modalities, 3D ultrasound correlated well with barostat volume measurements when assessing gastric accommodation [12]. Mundt et al. conducted a study to compare 3D ultrasound assessment of gastric accommodation with the barostat [31]. They found that almost all patients identified with impaired accommodation with the barostat were identified with 3D ultrasound [31]. The magneto-based system calculated gastric emptying rates more precisely than 2D ultrasound [10,27]. When choosing the division plane to assess separate gastric volumes, Gilja et al. applied a division line at the angular incisura due to easy visualization by ultrasonography [27]. In their opinion, division planes made ultrasonographically were more precise than those made with scintigraphy [27]. Mundt et al., however, felt that visualization of the angular incisura was difficult and, therefore, positioned their division line differently [31]. In conclusion, it seems that there is no established standard to correctly position division planes for calculating the intragastric distribution of meals [27].

Three-dimensional ultrasonography has many advantages when it comes to gastric measurements. However, it still has its limitations. The 3D ultrasound, like 2D ultrasound, could be influenced by air pockets and anatomical structures, which could affect visualization [15,27]. However, in one study, Gilja et al. did not have to exclude many images (2 out of 160) due to air pockets, and in another study, they managed not to exclude any of the images [23,27]. Mundt et al. also reported that up to 20% of air did not have any effect on the images [31]. Other adjustments to improve image quality were similar to the ones mentioned with 2D ultrasound, with the upright position and cooling of the meal. Another important general adjustment is to encourage the patient to burp if they feel the need to [11,27].

The 3D ultrasound imposes other challenges that are different from 2D ultrasound, such as the need for a magnetic shield and a tracking device [12,27]. This makes 3D ultrasound slightly less accessible [12]. Using a magneto-based system requires shielding from the surroundings due to its susceptibility to metallic influence and interference from external magnetic fields [11,27]. Influence from surrounding factors could impose distortion of data [27]. However, according to Gilja et al., the magneto system used in their studies is less susceptible than other magnetic modalities, and only a few of their images were affected [27]. Another challenge that is more prominent with three-dimensional ultrasonography is the time-consuming aspect due to the 3D reconstruction period [11]. The 2D ultrasound can also be time-consuming, but according to Gilja et al., evaluation at 20 min postprandially was good enough to gather sufficient measurements needed to assess proximal gastric accommodation [13,18]. Due to the added challenges with 3D ultrasound, 2D ultrasound has been more favorable to apply in clinical practice.

### 4.2. Future of Ultrasonography in FD Diagnostics

More recent studies on functional dyspepsia sought ways to differentiate the two subgroups, PDS and EPS. Pathogenesis seems to overlap between both groups and, therefore, has made the task more challenging [13,28]. A study by Ahmed et al. applied strain rate imaging to detect antral wall distension in both groups and found that EPS patients had higher antral strain measurements both fasting and postprandially than controls and PDS patients. EPS patients had hyper-contractility, while PDS patients were considered to have a hypo-contractility state of the antrum [5]. Strain rate imaging of the antrum could possibly be applied to discriminate between FD subgroups based on quantitative measurements of antral contractions [5]. Another study by Ahmed et al. focused only on PGA measurements in PDS patients [12]. Both of these studies give an insight into how the future of functional dyspepsia diagnostics with the incorporation of ultrasonography could become more personalized.

There has been a constant challenge to establish an association between FD symptoms and gastric motor disturbances [2,18,24]. Furthermore, we acknowledge that there are other pathophysiological mechanisms in FD, such as HP pylori, duodenal inflammation, and gut–brain interactions, that cannot be studied with ultrasound.

At Haukeland University Hospital, Bergen, Norway, ultrasonography has already been incorporated as a regular diagnostic procedure for FD patients, and this is called the *Ultrasound Meal Accommodation Test* [6]. UMAT has been applied at Haukeland University Hospital for more than 30 years and is still regularly used today [6]. UMAT enables sorting out dyspeptic organic conditions and simultaneously serves to assess gastric accommodation, contractility, gastric emptying, and visceral hypersensitivity [6]. UMAT provides not only information on gastric dysfunction in FD patients but also a psychometric questionnaire that allows for a more sound and personalized diagnostic procedure [6].

## 5. Conclusions

Functional dyspepsia is a common disorder seen globally, yet there are still big gaps in our understanding of its pathophysiology. Ultrasonography, being a non-invasive, safe, radiation-free, low-cost, and widely available method, has a huge potential for supporting FD diagnosis through the exclusion of organic dyspepsia and assessing FD pathophysiology. Incorporation of ultrasound in the work-up of patients with functional dyspepsia allows for a sound diagnostic approach and can further improve patient management. Despite its many advantages and technical capabilities, ultrasound appears to be underused worldwide in the care-taking of patients with functional dyspepsia.

## Figures and Tables

**Table 1:** Inclusion and exclusion criteria applied in this literature study on functional dyspepsia and ultrasonography.

Inclusion	Exclusion
Population that included those 18+	Population that included those under 18
FD condition present in population	Other conditions than FD, other conditions compared with FD, and other conditions amongst FD population
Ultrasonography as intervention	Other techniques
Outcome related to identifying/diagnosing FD	Other outcomes such as symptom relationships, therapeutic effects, etc.
English language	Non-English language

**Table 2:** Questions used for quality assessment of the articles included in this literature study on functional dyspepsia and ultrasonography.

Quality Assessment Questions
Does the article have a clear issue stated?
Is the method described well enough?
Is the study design appropriate for answering the issue stated?
Are the results appropriately presented?
Are the conclusions reliable?

## Data Availability

Not applicable.

## Appendix A

**Table A1:** This table presents the data extracted from the articles included in this paper, which were scientific studies that focused on the use of ultrasonography to identify functional dyspepsia pathophysiology. Author, year, country, aim of study, participants, equipment, technique, test meals, and patient position were extracted from these studies. The data clearly show that two main gastric parts were analyzed: the proximal and distal gastric parts. They also show that the ultrasound scanners and transducers applied differed and that several different techniques were used to assess gastric function. Different test meals were also applied, and patient’s position was mostly upright.

Author, Year, Country	Aim of Study	Subjects	Equipment	Technique	Test Meal	Position
N.K. Ahluwalia, 1996, UK	Gastric antral excursion and emptying	36 FD 25 HC	**Scanner:** Acuson-128, Acuson Computed Sonography, CA, USA **Transducer:** 5.0 MHz Linear Array	**Location:** Mid-line on upper abdomen to view cross-sectional antrum **Measurement:** Circumference of antrum	360 mL nutrient test meal with echogenic particulate material over 2–3 min	Upright
A.B. Ahmed, 2012, Norway	Antral radial strain in PDS and EPS	19 FD 15 HC	**Scanner:** System Five, GE Vingmed Ultrasound A/S, Horten, Norway **Transducer:** 6.7 and 5.7 MHz Doppler frequency	**Location:** 2 cm proximal to the pylorus, with the liver cranially and superior mesenteric vein and aorta posteriorly to view antrum **Measurement:** Antrum strain	500 mL meat soup over 4 min	Upright
I. Ahmed, 2023, India	Gastric accommodation	30 FD 30 HC	**Scanner:** Logic P6 PRO, Wipro GE Healthcare Ltd., Bengaluru, India **Transducer:** 2–5 Hz	**Location:** Longitudinally under left sub-costal margin and tilted cranially in the long axial direction to view sagittal section of proximal stomach **Measurement:** PGA	400 mL fat-free vegetable soup over 4 min	Upright
A. Berstad, 1996, Norway	Gastric emptying and motility, proximal gastric emptying, 3D volume	-	Proximal stomach: **Scanner:** CFM 750, Vingmed Sound, Horten, Norway **Transducer:** 3.25 MHz annular array 3D: **Scanner:** Vingmed Sound, Norway **Transducer:** 3.5 MHz	**Location:** Level of superior mesenteric vein and the aorta to view antrum epigastrium by the left subcostal margin, tilted cranially, and then rotated 90° clockwise to view proximal stomach **Measurement:** Cross-sectional antrum area, PGA, MPGD, PGV	Meat soup	Upright
L. Bolondi, 1985, Italy	Gastric emptying	34 FD 18 HC	**Scanner:** ALOKA SSD 250 **Transducer:** 3.5-MHz linear array	**Location:** Sagittal plane passing through superior mesenteric vein to view antrum **Measurement:** Cross sectional antrum area	800-cal Italian meal over 10–20 min	Both supine and upright position
D. Dorlars, 1994, Germany	Gastric Emptying	59 FD 50 HC	-	-	300 mL water	-
X. P. Fan, 2013, China	Gastric accommodation	45 FD 27 HC	**Scanner:** Voluson 730 **Transducer:** RAB 2–5 type	**Location:** longitudinally under the left subcostal margin and tilted cranially in the long axial direction, then rotated 90 degrees in the axial direction to view proximal stomach **Measurement:** PGA, MPGD, PGV	500 mL liquid meal (water + emulsion) over 4 min	Upright
O. H. Gilja, 1996, Norway	Visualization of antrum using 3D-US and antral volumes	20 FD 20 HC	**Scanner:** CFM 750, Vingmed Sound A/S, Horten, Norway **Transducer:** 3.25-MHz mechanical	**Location:** Abdominal skin **Measurement:** Antrum volume	Meat soup over 4 min	Upright
O. H. Gilja, 1996, Norway	Gastric accommodation	20 FD 20 HC	**Scanner:** CFM 750, Vingmed Sound, Herren, Norway) **Transducer:** 3.25 MHz annular array	**Location:** epigastrium by the left subcostal margin and tilted cranially in the long axial direction, then rotated 90 degrees in the axial direction to view proximal stomach **Measurement:** PGA, MPGD, PGV	500 mL Meat soup over 4 min	Upright
K. Haruma, 2008, Japan	Gastric emptying and antral motility	-	**Scanner:** SSA-260A, 270A; Toshiba, Japan **Transducer:** Curved array scanners with 3.75- or 5-MHz transducer	**Location:** Vertically to simultaneously visualize antrum, superior mesenteric artery and the abdominal aorta **Measurement:** Cross-section of antrum area	400 mL meat soup within 2 min Solid meal with chicken soup, rice and water over 10 min	Upright
T. Hata, 2013, Japan	Gastric motility and sensory function	26 FD 20 HC	**Scanner:** Aplio XV, Toshiba, Tokyo, Japan **Transducer:** 3.5-MHz convex-type	**Location:** 10th intercostal space using the spleen as an echo window **Measurement:** Cross section area of proximal stomach	800 mL water	Supine
D. Kamino, 2008, Japan	Long-term follow-up of antrum motor function	40 FD	**Scanner:** SSA-270A, 380A, or 390A **Transducer:** 3.5 MHz convex probe	**Location:** vertically to permit simultaneous visualization of the antrum, superior mesenteric artery, and abdominal aorta **Measurement:** Cross sectional area of antrum	400 mL consommé soup	Upright
M. Kato, 2010, Japan	Assess gastric motility and sensory function	60 FD 33 HC	**Scanner:** Aplio XV, Toshiba, Tokyo, Japan **Transducer:** 3.5-MHz convex-type	**Location:** 10th intercostal space using the spleen as an echo window **Measurement:** Cross sectional area of proximal stomach	800 mL water	Supine
T. Kugler, 2015, Ukraine	Gastric accommodation, emptying, and sensitivity	120 FD 30 HC	**Scanner:** Ultima PA **Transducer:** 1–5 MHz convex type	**Location:** - **Measurement:** Cross-sectional area of fornix	1000 mL water	-
H. Kusunoki, 2010, Japan	Gastric accommodation	44 FD 44 HC	**Scanner:** SSA-770A, Toshiba, Nasu, Japan **Transducer:** 3.75-MHz curved-array scanner	**Location:** Intercostal space of the left axilla, then vertically to permit simultaneous visualization of the antrum, the superior mesenteric artery, and the abdominal aorta **Measurement:** Cross-sectional area of proximal stomach and antrum	400 mL water	Supine
H. Kusunoki, 2000, Japan	Gastric motility	64 FD 36 HC	**Scanner:** SSA–260A, 270A, Toshiba, Japan **Transducer:** 3.75 or 5 MHz	**Location:** vertically to permit simultaneous visualization of antrum, superior mesenteric artery, abdominal aorta **Measurement:** Cross-sectional area of antrum	400 mL meat soup over 2 min Solid meal with egg and chicken soup with rice and water over 10 min	Upright
M. Loreno, 2004, Italy	Gastric emptying	20 FD 10 HC	**Scanner:** - **Transducer:** 5-MHz probe	**Location:** sagittal plane at the epigastrium passing through the level corresponding to the superior mesenteric vein **Measurement:** -	Solid meal of pasta, beef, bread, salad, apple, olive oil and water over 15 min	Upright
M.W. Mundt, 2006, Netherlands	Compare 3D US to barostat	15 FD 15 HC	**Scanner:** Esaote-Pie Medical, Maastricht, The Netherlands **Transducer:** 3.5-MHz curved probe	**Location:** Abdominal wall to localize stomach **Measurement:** Total gastric volume	500 mL Nutridrink + water over 3 min	Upright
